# Parent and perinatal professional priorities and perspectives for the pre-birth periviable conversation: a thematic analysis of semi-structured interviews

**DOI:** 10.3389/fped.2025.1552911

**Published:** 2025-07-01

**Authors:** J. Peterson, E. J. Johnstone, A. Mahaveer, D. M. Smith

**Affiliations:** ^1^ Faculty of Biology, Medicine and Health Sciences, University of Manchester, Manchester, United Kingdom; ^2^ Neonatal Intensive Care Unit, St Mary’s Maternity Hospital, Manchester Foundation Trust, Manchester, United Kingdom; ^3^ Maternity Services, St Mary’s Maternity Hospital, Manchester Foundation Trust, Manchester, United Kingdom

**Keywords:** neonates, extreme preterm, periviable, decision-making, trauma, maternity care

## Abstract

**Background:**

Periviable birth (22 + 0–23 + 6 weeks) presents clinicians and parents with numerous ethical, psychological and practical difficulties. The study aimed to identify key features within pre-birth periviable conversations for both professionals and parents, including priorities and challenges.

**Methods:**

Semi-structured interviews were conducted with participants from the key stakeholder groups: neonatologists/paediatricians (*n* = 5), obstetricians (*n* = 5), midwives (*n* = 5) and parents (*n* = 7). Interviews explored their experience of periviable counselling including priorities, challenges and perceptions. Thematic analysis was used to develop across parents and professionals.

**Results:**

Three themes were identified summarising the parent and professional perspectives within the pre-birth periviable conversations: chronology and narrative within pre-birth conversations, different perspectives on uncertainty and the role of transparency within periviable trauma. The trauma experienced by parents through periviable birth can be compounded through poor communication practices of perinatal professionals. These themes demonstrate that the information provided to parents should consistently outline all available care options relevant to their baby, including compassionately delivered, but honest and descriptive accounts of emotive options, such as comfort care. Information should be individualised to the specific circumstances and risk factors of that individual family and incorporate discussion of topics key to the ‘good parent belief’ to empower parents within their role.

**Conclusion:**

Perinatal professionals need to be able to utilise transparent communication, individualisation of information and understand the necessary role that narrative plays within decision-making. Future research is required to better understand the educational methods best suited to train perinatal professionals to incorporate these, and other trauma-informed care principles, within their communication and interactions with future parents.

## 1 Introduction

For infants born in the periviable period (22 + 0–23 + 6 weeks) there are high rates of death and neurodisability even with provision of intensive care (1). Due to the high mortality and morbidity rates, it may not be appropriate to offer active, survival-focused care to all periviable infants (1). The spectrum of potential outcomes makes pre-delivery discussions between clinicians and parents extremely difficult to conduct. A decision should be made between the perinatal professionals and parents facing periviable labour about the appropriateness of survival-focused or comfort care at delivery. In the case of comfort care, the infant is not subjected to invasive interventions at delivery and the focus of care is instead on ensuring the baby is comfortable, usually being held in their parents' arms, with the acceptance that baby will die in the delivery suite. Making this decision is intensely difficult for everybody involved. This decision-making process is further complicated by the increasing survival rates that have been seen in this cohort of infants following survival-focused care from specialist centres worldwide, with some centres reporting survival rates up to 80% (2). However, survival is not the only marker of the appropriateness of an intervention and there continue to be concerns regarding the additional risks of neurodisability with life-long learning and motor difficulties and respiratory, cardiovascular and metabolic complications which require consideration within the decision-making process (3).

Existing professional guidance and frameworks, such as the BAPM framework (2019) (1), attempt to assist clinicians in stratifying risk for extremely preterm infants and use this information to help guide the management decisions the perinatal professionals and parents face. Professional frameworks advocate for perinatal professionals to engage in a “shared decision-making process” with parents and to “convey information sympathetically and with clarity” (1). Current frameworks and perinatal professional training programs contain limited information about the structures, considerations and approaches that are needed to achieve these goals, making it problematic for professionals to actualise. Professional guidance is limited in detailing how these conversations can be crafted to ensure meaningful parental involvement without increasing parental trauma.

Trauma refers to experiences which cause intense physical and psychological stress reactions (4). This may be in response to an individual experiencing events or circumstances which they perceive to have a physically or emotionally harming effect on their physical, social, emotional or spiritual wellbeing (5). Psychiatric criteria for trauma describe extreme circumstances where the person is exposed to “actual or threatened death, serious injury, or sexual violence” (6). Whilst these circumstances may indeed result in trauma, the threat does not have to exist directly in relation to the person themselves for an event to be traumatic. This is reflected in alternative definitions of trauma which outline that trauma can occur in response to seeing threat to another person, rather than solely to oneself (5). The definition from Horowitz encapsulates this by stating that trauma can occur from “a sudden and forceful event that overwhelms a person's ability to respond to it” (7). This definition recognises that events can elicit trauma if they disrupt the individual's expected mental and life trajectory, or simply exceed the individual's ability to cope with that event (6). Periviable birth contains all the key features of a traumatic event due to the absolute disruption it brings to the parent's life, physically and emotionally (8).

Trauma-informed care (TIC) approaches have developed from research within populations at high risk of having experienced trauma, such as, people who have experienced multiple adverse childhood events (ACE's) (9). These approaches aim to avoid re-traumatisation and instead provide healthcare in an environment where the individual feels safe and can develop trust with the healthcare professionals (9). Trauma-informed care is being increasingly recognised in national healthcare plans and within the academic literature (5, 10–12).

There is increasing interest in how trauma-informed care can be implemented across different healthcare settings. Much of the previous work has been conducted in populations who have previously experienced trauma and the aim of the TIC approach is avoidance of re-traumatisation (8, 9). Whilst this is undoubtedly important work, the focus on TIC approach almost exclusively within groups who have experienced trauma in their past mean that those less familiar with the tenets of TIC approaches may perceive that this approach has little application to wider populations who may not have experienced severe or prolonged traumas, such as living through multiple ACE's, homelessness, severe mental health disorders. However, as is being increasingly acknowledged in national maternity reviews, such as the All Party Parlimentary review on birth trauma (12), interaction with maternity services and the process of giving birth can be traumatising events. The additional unexpected nature and significant threat to life that comes with periviable birth can compound this trauma (13). As demonstrated in the studies comprising this thesis, experiencing a periviable birth is a traumatising and life-altering event for parents. The trauma can be compounded by perinatal professionals through communication practices which perpetuate the loss of control and disempowerment experienced by parents. Compassionate care is a key principle within a trauma-informed approach, aligning with the principles of transparency, collaboration and empowerment (10). However, there are numerous high-profile maternity and perinatal reviews demonstrating a lack of compassion and care within maternity services (12, 14, 15).

## 2 Aim

This study aimed to identify and explore the key features, priorities and challenges within the pre-birth periviable conversation from both perinatal professional and parental perspectives. This information is required to identify how a trauma-informed approach can be integrated into these conversations to reduce the impact of trauma for future parents.

## 3 Methods

### 3.1 Patient and public involvement

During the design phase of the study the research team were able to meet with parents from the local parental advisory group (PAG) who had experienced periviable and extremely preterm birth and discuss the issue of how these conversations are conducted with parents presenting in periviable labour. Numerous parents within the PAG had lived experience of receiving poor communication from the perinatal professionals involved in their care. This ranged from professionals providing overtly contradictory information through to examples of striking lack of empathy and compassion from professionals. Parents reported these experiences were traumatic and detrimental to their ability to trust their perinatal teams. Parents in the PAG meeting agreed that the current approach to these pre-birth conversations requires rapid improvement and that empowering parents within these conversations and providing them access to reliable information should be priority issues for neonatal research agendas.

Following on from the initial PAG meeting, two PAG members were appointed as parent representatives for the study to assist with study development and act as a point of contact between the research team and the PAG going forward. These parent representatives had lived experience of periviable birth. Through conversation with the parent representatives, it was determined that semi-structured interviews would be useful and acceptable method to investigate the priorities and challenges experienced by parents and professionals within these pre-birth conversations. The aim would be for equal representation across parents and perinatal professional groups through similar numbers of each being recruited for interviews. Parent representative feedback was sought on the developed semi-structured interview topic guide to ensure that the included questions were acceptable to parents and phrased sensitively. Additionally, one of the parent representatives was able to participate in the pilot interview to trial the topic guide and cross-check that questions were being sensitively phrased and responded to by the interviewing researcher. Additionally, parent representatives were able to check public-facing documents, such as recruitment letters and posters.

### 3.2 Study design

The study has been developed and reported in accordance with the consolidated criteria for reporting qualitative studies (COREQ) checklist (16). The survey received favourable ethics review from the North West—Liverpool Central Research Ethics Committee (22/NW/0052).

Semi-structured interviews were selected to gather in-depth perspectives from perinatal professionals and parents with lived experiences of periviable birth. The semi-structured interview topic guide was developed by JP based on past literature and her expertise. It was cross-checked by DMS and by the parent research representatives who distributed the guide to the wider PAG for feedback. This process ensured that the interview topics were checked for sensitivity and adequately addressed the parental perspective and research aims (17). Two pilot interviews were conducted prior to starting recruitment; one with a parent representative for the study and one with a professional. Neither of the pilot interviews were included in the analysis.

Interviews were conducted either face-to-face or via video call using Microsoft Teams at the preference of the participant. A well-defined distress protocol was developed, in collaboration with the counsellors from the neonatal unit and the maternity and neonatal bereavement teams. The interview was audio recorded using a Dictaphone and then the recording was transcribed verbatim by a hospital approved medical transcription company (Accuro) (18). The transcription company selected (Accuro) (18) are a well-established and NHS approved transcription company who have expertise in medical transcription. They utilise a secure platform to transfer data between the study site and the transcription service. Their staff specialise in medical transcription and the company have their own psychological support services available to their staff who listened to and transcribed the recordings. This was particularly important in relation to this study as these audio recordings contained material that was emotionally demanding.

The interviews were all conducted by JP who is a clinician with experience in delivering bad news and dealing with emotional distress. If the parent or clinician experienced emotional distress, the option was given to pause or stop the interview, and the distressed participant could have some time to recover or discuss the distressing topic with the interviewer. Parents whose child had died had pre-existing access to the midwifery or neonatal bereavement team who could also be contacted for support. If the distress was profound or sustained, support was sourced from the neonatal counselling service. All interviews were scheduled at times when the counsellors were available in case they were required. JP also provided the contact details of the counsellors to the participant at the end of the interview in case they wished to contact the counsellors at a later stage. The option for the interview to be conducted via online video call avoided parents having to attend hospital as this had the potential to be distressing.

After the interview, participants were sent a £20 gift card as a token of appreciation. The transcribed interviews were anonymised and electronically stored under a coding system. The anonymised interviews were then analysed by JP and DMS using the Braun and Clarke six-stage reflexive thematic analysis process (19).

### 3.3 Study setting

This study has been conducted across a multi-site maternity service in the North West of England which has approximately 17,000 births annually. This service is comprised of a specialist maternity and neonatal unit (Level 3) with intensive care and surgical facilities that regularly manage extremely premature infants from 22 + 0 weeks gestation, and two local maternity and neonatal units (Level 2). All three units work in close collaboration and have experience in conducting periviable counselling conversations with parents. Where possible, it is preferable for mothers in threatened preterm labour to be transferred to a tertiary unit. However, the maternity and neonatal teams at the local (level 2) units would have experience conducting the pre-birth conversations with parents presenting in threatened periviable labour and have experience providing stabilisation to periviable infants where there was not sufficient time for an in-utero transfer to a specialist centre. It was important to ensure recruitment of perinatal professionals from both specialist and local centres to capture potential variation in attitude, approaches and priorities between professionals who more regularly manage periviable infants (specialist unit) and professionals who may have limited exposure to these infants (Level 2 units).

Given that the focus of these interviews was on information-sharing practices in relation to decisions made regarding management of the periviable infant, the research team decided to recruit from perinatal professionals who were most likely to be senior decision-makers and to have the most experience of these conversations. Therefore, study recruitment was limited to consultant obstetricians, consultant neonatologists, consultant paediatricians and experienced midwives (Table 1).

**Table 1:** Inclusion and exclusion criteria.

Inclusion criteria for parents	Exclusion criteria for parents
Recent experience of periviable counselling (within 24 months of the study being open to recruitment)	Parents were not eligible if they did not have parental responsibility and if they did not speak, read and write English
Periviable gestation when pre-birth counselling conversation took place (22 + 0–24 + 6 weeks)	Parents were not eligible if the pre-delivery counselling conversation occurred more than 2 years prior to the study being open to recruitment
Parents were eligible if they had a periviable counselling conversation, regardless of whether they then delivered or not and regardless of whether the delivered infant was not alive at delivery, died in the delivery room, died on NICU or survived to discharge	Parents were not eligible if the pre-delivery conversation occurred when they were <21 + 6 weeks or >25 + 0 weeks gestation
Parents must have parental responsibility for the child	
Parents must be able to speak and read English fluently. There was no funding in this study for translation services	
Inclusion criteria for professionals	Exclusion criteria for professionals
Clinicians were eligible if they were a Consultant Obstetrician, Consultant Neonatologist, Consultant Paediatrician or Midwife.	Not within the specified perinatal professions (Consultant Obstetrician/Neonatologist/Paediatrician or Midwife)
Employed and working within one of the hospitals in the designated multi-site maternity service in the North West of England	Not employed within the designated multi-site maternity service
Clinicians must be able to speak and read English fluently	

### 3.4 Recruitment

#### 3.4.1 Parents

Parents will be identified by the research team from neonatal electronic record [Badger.Net (20)] and the bereavement team records. The research team screened against the pre-determined inclusion/exclusion criteria (Table 1). Parents were contacted in sequential order working back from the most recent. Parents of surviving infants were contacted by post by the research team. Bereaved parents were contacted via their bereavement team at an appropriate moment during routine face-to-face or telephone follow-up and would only be contacted if the death had taken place more than 12 months ago. Parents were included if their baby had been born between 22 + 0–24 + 6 weeks to reflect that the periviable period evolves over time and whilst survival-focused care is now considered from 22 weeks in the United Kingdom, professional guidelines (released in 2019) continue to include infants born at 24 weeks within the “High Risk” group for death and disability (1).

#### 3.4.2 Professionals

Consultants were identified by the research team through the hospital staff listings on the Trust internet page for the included consultant professional groups listed in the inclusion criteria. From these staff listings, the research team assigned a number to each professional. For each professional subgroup, a random number generator was then used to select which individuals to contact to invite to participate in the study. In cases where a professional declined to be involved their number was removed, and another randomly generated individual within that subgroup would be contacted. The staff listings were separate for the tertiary and local perinatal hospitals in which this research was conducted. Therefore, it was possible to ensure that recruitment of consultant neonatologists and obstetricians was approached equally across tertiary and local units by applying the random number generator stepwise across each staff list within each specialty.

Midwives were recruited through displaying recruitment posters in delivery suite and the postnatal wards and emailing an electronic version of the poster to midwives across the three recruitment hospitals.

Participants were recruited to participate in one interview. All participants had to provide written consent. The transcripts were not shared with participants, but participants could opt to receive a summary of the study findings from the research team at the end of the study.

### 3.5 Study size and analysis

The study was granted ethics approval to recruit and conducted up to 30 semi-structured interviews, comprised of a maximum 15 parents, five consultant obstetricians, five consultant neonatologists/paediatricians and five midwives. This composition was selected to represent the perspectives of the multidisciplinary team involved in caring for parents experiencing periviable delivery. Interviews were transcribed and analysed continually and when thematic saturation occurred (defined as no new themes being identified in the data from two consecutive interviews) then recruitment was stopped.

The anonymised interviews were then analysed by JP and DMS using the Braun and Clarke six-stage reflexive thematic analysis process. The electronic transcripts were initially coded independently. The two researchers (JP and DMS) then met to determine and refine the identified themes through a series of discussions.

### 3.6 Researcher reflexivity

Researcher reflexivity statements are provided for the two researchers (JP and DMS) who directly conducted the interviews and/or the reflexive thematic analysis.

JP conducted all the interviews and analysed the transcripts using reflexive thematic analysis. She occupied a dual role of both “insider” and “outsider” within different dimensions of the research. JP is a senior neonatal subspeciality trainee and research fellow and had worked within the centres that the research was conducted in. Her professional role provided her with insight into the medical and logistical issues involved in periviable birth and her clinical experiences observing consultants conducting these pre-birth conversations allowed her to appreciate the variation in information being shared with parents and led to her developing the research question and this study. This knowledge of periviable birth and her familiarity with the recruiting sites placed her in an “insider” position. This insider knowledge provided familiarity with organisational process and provided useful insights from which a robust and sensitive recruitment process, interview topic guide and distress protocol were developed (see Supplementary Files). Her position as an “insider” had the potential to confer disadvantages during the interview process and this position allowed her to understand implied content from participants. This issue was identified by the supervising researcher (DMS) during the pilot interview and allowed JP to ensure in the subsequent interviews that participant responses were unpacked verbally through follow-up questions, including on topics with which JP may have been familiar due to her professional role. Her role as an “insider” was acknowledged at the beginning of the interviews with participants for transparency. This role as a practicing neonatal professional could have been a barrier to parents fully expressing their views on the care they received from perinatal professionals. This is addressed through JP outlining that she and the wider research team were aware that current information sharing practices with periviable parents were imperfect and that the motivation for this study was to understand the issues within current practice, in order to identify how best to improve care for future parents facing periviable birth. Within her professional role, JP does not hold a consultant position. This provided her with an “outsider” perspective which allowed her to identify the variation in information shared with parents as an area requiring research and provided her with a level of objective detachment when performing the thematic analysis.

DMS is the supervising researcher and conducted reflexive thematic analysis of the interview transcripts. She is a psychology lecturer and an established qualitative researcher, with particular expertise in maternity qualitative research and an interest in stillbirth. DMS broadly occupied an “outsider” role within this study. This provided her with objectivity regarding the thematic analysis and insight into identifying issues of implied content for JP within the pilot interview. DMS has personal experience of being a parent on a neonatal unit, not related to periviable or extreme prematurity. This, and her previous research on stillbirth, places her in the position of “informed outsider”. There are potential risks both from “insiders” conducting research (for example, comprehension of implied content during interviews) and “informed outsiders” conducting research (for example, possibility of projecting biases and personal experiences onto the data) (21, 22). By having both the “insider” and “outsider” role represented within the research team and engagement from both in serial discussions about the interview data throughout the analysis phases (generating themes, reviewing themes, defining and naming themes), the researchers were able to discuss each perspective and ensure the identified themes were objective and derived solely from within the interview data itself.

## 4 Results

A total of 22 semi-structured interviews were conducted. These comprised 15 interviews with perinatal professionals, with an equal split across obstetric, neonatal/paediatric and midwives, and seven parents, four of whom had a surviving child and three parents whose baby had died. Of the seven parents, there were six mothers and one father. One set of parents opted to be interviewed together. All parents spoke fluent English with English being the primary language for five parents and an additional language for two parents. There were a range of ethnicities and socioeconomic backgrounds represented across the interviewed parents. All the interviews with parents were conducted via online video call. All interviews with consultant obstetricians and midwifery professionals were conducted via online video call. For consultant neonatologists and paediatricians, three interviews were conducted face-to-face and two were conducted via online video call at the preference of the participant. The parents of surviving children had experienced periviable birth between 22 + 2–23 + 6 weeks. For the parents whose baby had died, their baby had been born between 24 + 0–24 + 6 weeks gestation and had died on the neonatal unit.

The parent interviews lasted for an average of 64 min [mean = 64 min; standard deviation (SD) = 14 min]. Perinatal professional interviews lasted a similar length of time with an average 61 min (mean = 61 min; SD 10 min). All interviews proceeded without needing to utilise the distress protocol.

**Figure 1:**
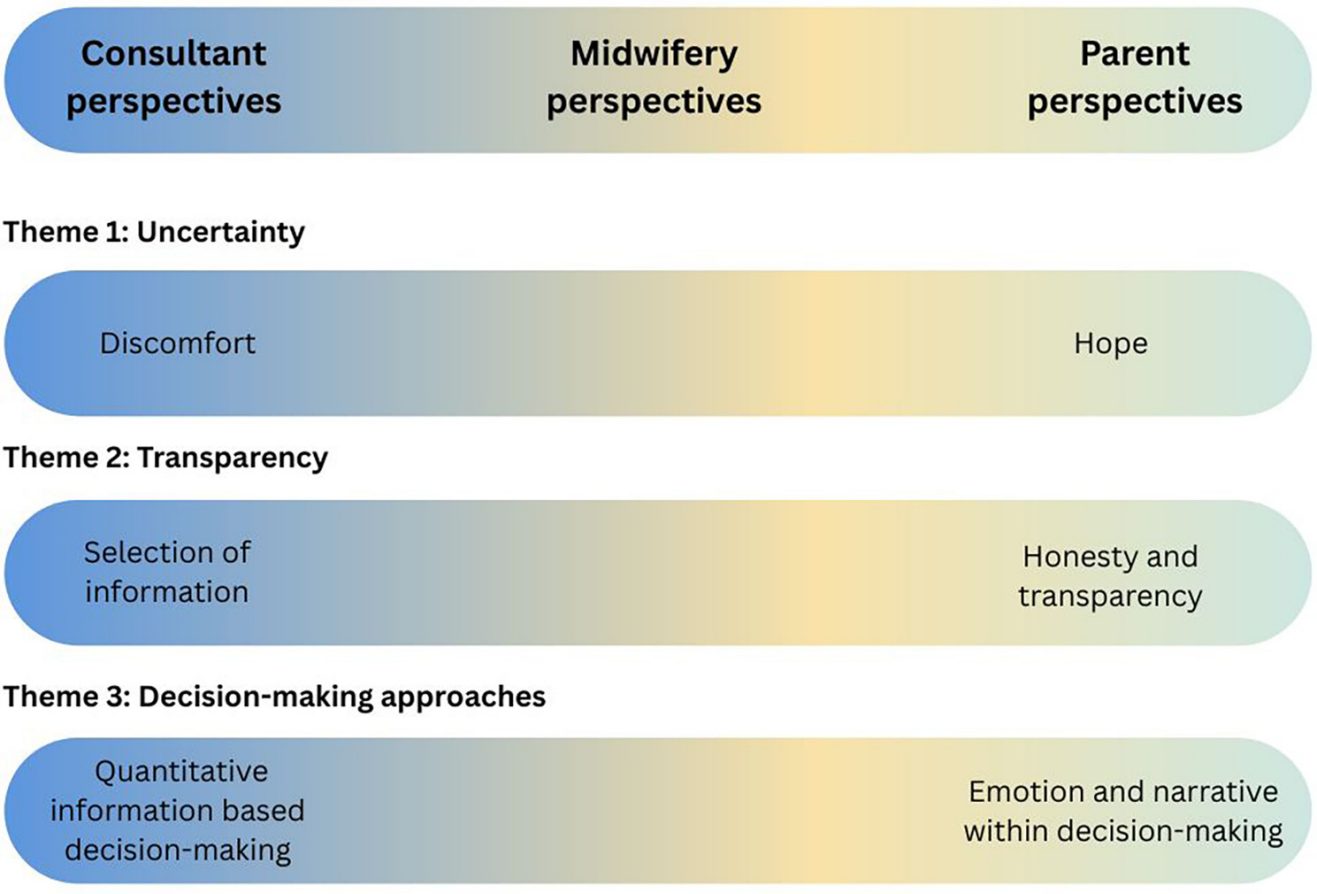
Overview of identified themes.

From the interview data three themes were identified (Figure 1 and Table 2). These were 1. Uncertainty, 2. Transparency and trauma and 3. Narrative within periviable decision-making. Whilst these themes were present across both parent and professional responses, they were often expressed as counterpoints to the other's perspective. These opposing perspectives and narratives have potential to create distance and disengagement, or even conflict, between parents and professionals. The midwifery professionals occupied a unique vantage point across the themes, with their responses witnessing both parent and consultant perspectives and reflective of both.

**Table 2:** Illustrative quotes by developed theme.

Theme 1: Uncertainty		
Consultants	Midwives	Parents
“I…probably a lot of people find [pre-birth periviable conversations] uncomfortable… because it's a very…it's…they are very difficult conversations and there's no absolute definites”	“We're very lucky to have the facilities and the expertise there but despite all that sometimes we can't intervene and make problems better and that's the reality of it unfortunately.”	“…[the stats] still don't picture a very good light on it because a lot of babies across the country still aren't saved at 22 weeks so to have a success rate on there, I think it says something like 10%. But if the majority of babies are refused at 22 weeks, then the success rate is never going to be accurate is it?”
“I feel that we don't always know. We have babies in follow up clinic, who had grade 4 IVH's and VP shunt and they're doing great, so I don't feel in a position that I can say for sure I know that this is going to be terrible because sometimes it's not.”	“I think it's a challenge that the more you're exposed to it the more you become the realisation is you can't fix it all although we try to.”	“….we know if we get to 22 weeks she's got a chance here”
“I think we have to think about what's right and what's just trying to push things to a limit.”	“I suppose feel a bit of acceptance that we can't make everything better.”	“She [NICU Cons] did say that if she had a baby born at this gestation that she wouldn't want that baby to do anything because the baby would need that much care. So it was a personal opinion put on it as well. So, to us, it was, let's take that risk.”
“…are we saving a life, or deferring a death” “…we get them to the neonatal unit … that's wonderful but I think it's the complete package of what you're looking at for the rest of your life and for that family"	“My experience has just been we're going to do everything it's never been let's break it down and see what everything means it's been almost that the shutters are down we want everything done conversation finished. That's been it.”	“I think because we weren't 100% sure how it was going to go with the birth …we discussed if she does survive she is going to be on a hell of a lot of help because of her size, because she's premature and we said like we'll just go with her … at the end of the day the only one that can do what they've got to do is her, once she's out it's up to her.”
“Always in the back of our mind is, are we going to carry out a delivery where it's going to be so complex that she's going to need to have a hysterectomy and then that completely ruins her chance of having any more children in the future? That is one of the most difficult conversations the obstetricians can have I think personally, whenever babies are very preterm.” “…it's a difficult balance, isn't it, of doing the best by the pregnancy that is immediately in front of you versus always kind of having that eye on future pregnancies…”	“…as professionals with our own exposure and experience to these babies and I'm sure everyone will have the good and the bad outcomes and it's difficult for that not to cloud your judgement when you are giving advice”	“It came across that nobody just believed that it could happen. So I don't really know. I just, I think there was just that belief that the baby wouldn't survive and therefore there was no point trying, that's what made us cross”

Theme 2: Transparency		
Consultants	Midwives	Parents
“I would wait for the parents to raise it [possibility of comfort care].” “I wouldn't do that [discuss comfort care]. I mean 23 weekers can do well, absolutely fine, and how do you just give them an option of elective comfort care?”	“…if you want [parents] to be more involved then you can't be cherry picking what the information is we're providing … we need to give it them all…they [the doctors] think they're protecting people…but they're not”	“That [information about comfort care] would really help as well like, let's say some mums would be positive about their kids who have probably passed like they can talk about it and you could read information on that. Maybe that might help you as well just in case something did go wrong…Instead of all of it being really positive, I think you do need that reality check sometimes.”
“It's individualised depending on what you know the parents want to happen.”	“You're making assumptions that you're telling them what you think they want to hear and that's not necessarily the case.”	“…moved up to delivery and the neonatal doctor came in himself, he was very very good [NICU Cons]. He was absolutely amazing he literally went through everything”
“Probably lean slightly on the…lean slightly on the paternalistic because I wouldn't go through every single option with them …”	“I think it's okay to say we don't know and I think sometimes we are scared to say we don't know or we are worried that it makes us look incompetent or something like that….but I think it's honesty. It's just transparency. It's okay and actually, for me, I'd be more likely to trust a doctor who was honest with me than if I felt they were hiding something.”	“having all the information to hand and letting mums know that may have a premature birth is quite important, it is essential, I didn't even know about it. The different types of unit…. I didn't know about them.”
“…with older gestations [23 weeks], then I get a bit uncomfortable giving certain options outright to say not do anything.”	“I feel the families, the more information they've got they feel better equipped to make these decision when they are being involved”	“And what we found is a lot of this information was actually available on NICU after the baby had been born and it was sort of well I know all this now. It just seemed a bit backwards.”
“I don't think that I specifically talk about the spectrum of disability in antenatal counselling. Unless I guess …I might say a third might have some disability, a third won't and then I would, if they pushed me within that, then I'd explain more about that I guess.”	“I think that is the most important thing that they can say to someone. I think that is just completely managing your expectations, you know like, we'll try our best, but there's no guarantee that that will be possible and I think sort of emphasising that your baby is very early and that your baby often is quite small makes these families understand a little bit more that yeah, do you know what, they're not lying to me. They are telling the truth and I think that's really important.”	“So with [tertiary unit] we had all information, every single time we were there, every single time…They would say this is what I'm doing now, this is why I'm doing this and this is what is happening next and if your baby reacts this way, this is how we will want to see him, so we definitely knew what they were doing. We knew who we were talking to, we knew what was happening and they were always signal our concerns, so it was really very easy to be in [tertiary unit] with [Baby's Name] and so we are so very happy with the care we received there.”
	“…it's okay to say we don't know and it's okay and doctors don't know everything either. They're not godlike.”	“…. we had a lot of regrets after losing [name of previous child who died at periviability]. We felt like, you know, you'd wake up in the middle of the night and think, why didn't I do that differently? Or you know, you put a lot of blame on yourself, and I think there was a lot of conversations that we had that made us think, well what could we have been done differently and you know what other options would there be…”
		“To be honest my time in triage …was my most traumatic time because of the gestation of the pregnancy I felt like the doctor that I spoke to completely wrote [the baby] off…”
Theme 3: Narrative around periviable decision-making		
Consultants	Midwives	Parents
“I would have discussion with parents based on the BAPM [British Association of Perinatal Medicine] standards, based on our statistics… what you're going to take into account the history of the mother's history, like you say chorioamnionitis, growth retardation and all those kinds of things and how we're going to pitch it…”	“The parents were listened to which was massive.”	“Some [professionals] were saying they can save babies at 22 weeks…we save babies at 23 weeks and then someone else was telling me 24 weeks.”
“I think you kind of owe it to them [the parents] to kind of lead them in a sense, and obviously it doesn't come down to, and it should never come down to, what would you do in this context? Because you can never know. Because if it was you in that context, then of course, then you'd have the effect of the emotional stuff and all the rest of it, and I wouldn't want to make a decision for myself in that way.”	“I know reflecting on a particular encounter has involved an obstetrician at a smaller centre she kept referring to her own experiences with her own delivery of a very pre-term child that she'd had and I didn't feel that was necessarily the most appropriate conversation to be having she should have kept it more personal to the patient as opposed to herself"	“You know, it's not a night we'll forget very easily. It was, it felt like we were battling the hospital.”
“There are some [surviving] babies that are out there but the numbers are small…”	"The midwives can explain [comfort care] to the families in a way that they're much more likely to understand but also to sort of find some peace with rather than …often when you see some doctors speak to them, the way that it's phrased … I think it's all just about terminology…”	“…we knew if [baby] was delivered that night, they wouldn't have done anything….so we needed the time, we needed the agreement, we need NICU to be there…”
“…we've been offering support to 22 weekers on the basis that [senior tertiary NICU Consultant] always said, it's a self-fulfilling prophecy. If you say they always have poor outcomes, you don't attend to them and they all die well, yeah, then they all die. So, we don't actually know what the outcomes are.”	“The situations that I can recall have been they've actively done everything and some, where, unfortunately, it's been unsuccessful ….so I've seen both sides neither of those decision situations are positive outcomes unfortunately but for the families they know … in their minds “everything” was done and their wishes was respected I think that was the biggest sort of lesson from that ….The families felt like their wishes were carried through.”	“Sometimes you do try to be realistic about things, but you still want that positive reassurance. Obviously with doctors it's more of we're giving you the facts. We don't want to give you false hope - which I understand - but sometimes you just wish you could have that false hope…. Obviously some of them would give me the information that I didn't want to hear but they were really nice in the way that they delivered the information.”
“ …there are those parents who usually want everything doing insofar as - when I say “everything” doing…. what I actually mean by “everything” is to offer [breathing] support at delivery and see how they respond.”		“…it probably was just enough that they [NICU] were going to come and they were going to do something. What that something was going to be, I knew depended on the baby, but we didn't know a lot of what they were going to do.”
		“They [the NICU doctor] did mention something like obviously if she's breathing they will help her and stuff like that and then if she wasn't then that would be it … but I just remember saying just do everything that you can. They did say that her brain could be affected, she could be disabled and she could be not able to breathe for herself for a while.

### 4.1 Uncertainty as a source of hope, acceptance or discomfort

“There are so many uncertainties” [Consultant Paediatrician]

The inherent uncertainties which surround periviable birth were acknowledged within both parent and professional responses.

The parent responses demonstrated an acceptance that uncertainty was an intrinsic part of periviable birth, and that this uncertainty could contain hope; “*She [NICU Cons] did say that if she had a baby born at this gestation that she wouldn't want that baby to do anything because the baby would need that much care… To us, it was, let's take that risk*.” [Parents of surviving 22 week baby]. Others framed this uncertainty through the construct of the baby's autonomy; “*I think because we weren't 100% sure how it was going to go with the birth and that really …. we had said like we'll just go with her [daughter]—at the end of the day…once she's out it's up to her*.” [Parents of surviving 23 week baby]. This was a strategy for managing uncertainty which was also utilised by some professionals; “….*we would just be guided by the baby*…” [Consultant Neonatologist].

For professionals, one of the key components generating uncertainty was the evolving nature of outcomes for these infants and that advances in perinatal care may mean that the prevailing professional perception of outcomes may lag behind the reality; “*part of the problem is, is that we don't actually know the specific numbers… and we certainly don't know the specific numbers in the specific unit to any updated, reasonable degree*.” [Consultant Neonatologist] and, “*I think first of all we need to get a bit more educated by the neonatal team as to what the realistic picture is in today's modern neonatal unit*.” [Consultant Obstetrician].

The presence of uncertainty was presented with discomfort by both obstetric and neonatal/paediatric consultants; “*I…probably a lot of people find [pre-birth periviable conversations] uncomfortable*. *because it's a very…it's…they are very difficult conversations and there's no absolute definites*” [Consultant Paediatrician]. This led to statements from professionals which contained internal shifts between the optimism and pessimism that can exist within areas of uncertainty; “*Like I say because it's not…I've never met a 22/23 weeker that's not had some kind of issue like they usually go on home oxygen, on NGT [nasogastric tube ] feeds….may need kind of like physiotherapy and things like that….At the same time some of them have these little things like home oxygen but they are off oxygen in a few months, it's a bit…difficult*” [Consultant Neonatologist].

Other consultants described the potential for uncertainty to exert a negative effect within the infant's management, describing uncertainty as a “trap”; “*…and there is always that worry that…you kind of get into a position where you lose and start to tip a little bit, you know, and you go well how come we've ended up here…*” [Consultant Neonatologist], and,

“*I suppose you're trapped in the sense that once you've put a baby on a ventilator and you're doing gases every few hours and your team are responding to that, then it becomes natural [to continue intensive care]….* *it feels like a conveyor belt kind of spinning away underneath you….It's just a nigh on impossible task because ultimately we can't be 100% sure at any point which babies are going to pull through and which are not*.” [Consultant Neonatologist].

All consultant responses contained numerous instances of consideration of the impact of periviability over the lifetime of the child and family, such as, “*…we get them to the neonatal unit … that's wonderful but I think it's the complete package of what you're looking at for the rest of your life and for that family*” [Consultant Neonatologist], and provided examples illustrating concern that provision of intensive care to these periviable infants led to a prolonged death, rather than necessarily to survival; “*…are we saving a life, or deferring a death*” [Consultant Neonatologist].

For the consultant obstetricians, the role of time was particularly problematic to navigate. From the interview data, a core component of the obstetricians' self-perceived role was in maintaining a balance of assisting, and optimising where possible, the current periviable pregnancy, against consideration of their patient's reproductive future; “*it's a difficult balance, isn't it, of doing the best by the pregnancy that is immediately in front of you* versus *always kind of having that eye on future pregnancies…*” [Consultant Obstetrician], and,

“…women that are 23 weeks, where you're doing everything possible, and they more likely have a breech presentation at that gestation, they automatically know that caesarean sections are what happens whenever your baby is breech and therefore that’s what they expect and that can sometimes be difficult to reconcile yourself to. Is this the right thing to be doing? Is this going to improve the outcome for that baby? Is it going to cause significant trauma to the mother? Always in the back of our mind is, are we going to carry out a delivery where it’s going to be so complex that she’s going to need to have a hysterectomy and then that completely ruins her chance of having any more children in the future? That is one of the most difficult conversations the obstetricians can have I think personally, whenever babies are very preterm.” [Consultant Obstetrician].

The presence of uncertainty was also noted within the midwifery responses, however, these contained an acceptance of uncertainty; “*I think it's a challenge that the more you're exposed to it the more you become the realisation is you can't fix it all although we try to.”* [Midwife].

### 4.2 Transparency and trauma

Periviable birth is a traumatic event with this trauma being experienced as loss of parental control through the, often unexpected, occurrence of periviable birth and through the uncertainty and fear that exist for parents who have a critically ill or dying baby. Within this traumatising event, there can be additional layers of iatrogenic trauma from suboptimal or ill-considered communication approaches from perinatal professionals.

Throughout the parent interviews there were clear references to the trauma of the experience being compounded through negative interactions with perinatal professionals; “*To be honest my time in triage*…*was my most traumatic time because of the gestation of the pregnancy I felt like the doctor that I spoke to completely wrote it off*…” [Parent of 23 week surviving baby]. Parents utilised language of having to fight for access to information and for their baby to have access to care; “*You know, it's not a night we'll forget very easily. It was, it felt like we were battling the hospital*.” [Parent of 22 week surviving baby].

Parents encountered significant pessimism from perinatal professionals in relation to periviable infant outcomes, including from the point of presenting to the hospital and being triaged; “…*[the midwives at triage] directly took us to the bereavement room. We didn't know we were going there, there was no discussion about it*” [Parent of 22 week surviving baby]. This immediate pessimism from professionals created a sense for parents that the life of their baby was being dismissed; “*I said save the baby because he's valuable. You know after losing my first baby I started to do all sorts of research and I said my baby's valuable and with the right care he can make it through so I said my baby's valuable….”* [Parent of 24 week baby who died]. These dismissive interactions with professionals can compound the loss of control and isolation that parents may experience during a periviable birth and leaves parents feeling they are having to “fight” professionals, rather than being supported by them; “*….we felt like we were just battling with every single person that we’d met.*” [Parent of 23 week surviving baby].

In instances where parents felt they had received limited information or that their baby had not been adequately considered for survival-focused interventions, such as antenatal steroids, this had a lasting impact on parents and evoked feelings of regret; “*[in relation to care of their previous baby who died at periviability]…. we had a lot of regrets after losing [name of previous child]. We felt like, you know, you'd wake up in the middle of the night and think, why didn't I do that differently? Or… what could have been done differently and, you know, what other options would there be…*.” [Parent of 22 week surviving baby].

The parent responses all indicated that parents valued two things: 1. access to a full picture of information which was relevant and individualised to their baby's circumstances, and 2. compassion and engagement from professionals with their baby as an individual. Parents did not want professionals to mask or omit sensitive or distressing topics. Rather, parents were accepting that they needed to be informed of all applicable management options and risks, even if these were difficult to hear; “*That [information about comfort care] would really help as well … let's say some mums would be positive about their kids who have probably passed… like they can talk about it and you could read information on that. Maybe that might help you as well just in case something did go wrong….Instead of all of it being really positive, I think you do need that reality check sometimes*.” [Parent of 23 week surviving baby].

There were additional systemic factors which served to compound the sense that information was being hidden from parents. These included numerous parents highlighting that there is little to no information provided about preterm birth during their pregnancy despite it being a relatively common complication. Parents also emphasised that the structure of hospitals and neonatal networks is not discussed with expectant parents, for example, presentation in extreme preterm labour at a local hospital may necessitate transfer to a different specialist hospital, and, therefore, this information comes as an additional shock for parents facing extreme preterm labour, further increasing their sense of loss of control: “*I was taken to [specialist hospital] … it felt like a million miles away*” [Parent of 24 week baby who died].

Responses from consultants showed variability in approaches in discussing options and outcomes with parents. There were clear examples of consultants utilising professional frameworks to individualise the information they were providing to parents, and others who used functional outcomes and milestones to make discussion of long-term outcomes tangible for parents;

“I think working through [professional framework] and taking a more individualised approach, looking at the gender of the baby, the birth weight of the baby and what the parents' wishes are has been helpful in trying to develop a more individualised approach, because it's definitely not one size fits all when it comes to these kinds of discussions.” [Consultant Paediatrician];

“I'd always just say that it's a spectrum and that. So the spectrum might look like at one end of the spectrum it might be a child that's in some of mainstream school that might need some help in terms of, perhaps they might have some dyslexic tendencies and perhaps need some additional input, but within the context of a mainstream school and they might be able to walk and talk and do all the other things that they need to do in terms of their own independence. And at the other end of that extreme is a child that can't do anything independently for themselves. So they may well not, they might not be able to walk. They might be dependent, therefore, upon a wheelchair, might not be able to feed themselves. They might not be able to express themselves with words. They might not ever be able to live an independent life essentially.” [Consultant Neonatologist].

Conversely, other consultants expressed concerns about overwhelming parents with information and described a process of selecting information about options and outcomes that they would present to parents, or, in some cases, would only discuss with parents if the parent specifically asked: “*I wouldn't do that [discuss comfort care]. I mean 23 weekers can do well, absolutely fine, and how do you just give them an option of elective comfort care?*” [Consultant Paediatrician], and “*I would wait for the parents to raise it [possibility of comfort care].*” [Consultant Neonatologist]. This phenomenon of information being selected for presentation was represented within the midwifery responses where it was highlighted as an area of concern; “…*if you want [parents] to be more involved then you can't be cherry picking what the information is we're providing … we need to give it them all….they [the doctors] think they're protecting people….but they're not*” [Midwife]. The midwifery responses identified that acknowledgement of uncertainty and transparency of information provision were essential elements of building trust with parents: “*I think it's okay to say we don't know and I think sometimes we are scared to say we don't know or we are worried that it makes us look incompetent or something like that….but I think it's honesty. It's just transparency. It's okay and actually, for me, I'd be more likely to trust a doctor who was honest with me than if I felt they were hiding something*”. [Midwife].

### 4.3 Narrative around periviable decision-making

Within the interview data, the fluctuating ways that the narrative around the birth was constructed were varied across both the parent and professional interviews. Within the professional interviews there was an acknowledgement that provision of intensive care to the periviable infant is an emerging area of medical advancement with, as yet, no established evidence base for the optimal management of these infants set against the certainty that not intervening after birth will lead to death; “*…we've been offering support to 22 weekers on the basis that [senior tertiary NICU Consultant] always said, it's a self-fulfilling prophecy. If you say they always have poor outcomes, you don't attend to them and they all die well, yeah, then they all die. So, we don't actually know what the outcomes are.”* [Consultant Neonatologist]. Despite this, the consultant responses revealed a predilection for factual, quantitative information with which to guide their decision-making and a relegation of emotion within the decision-making process;

“I would have discussion with parents based on the BAPM [British Association of Perinatal Medicine] standards, based on our statistics… what you're going to take into account the history of the mother…chorioamnionitis, growth retardation and all those kinds of things and how we're going to pitch it…” [Consultant Neonatologist]

“I think you kind of owe it to them [the parents] to kind of lead them in a sense, and obviously it doesn't come down to, and it should never come down to, what would you do in this context? Because you can never know. Because if it was you in that context, then of course, then you'd have the effect of the emotional stuff and all the rest of it, and I wouldn't want to make a decision for myself in that way.”. [Consultant Neonatologist]

Conversely, parent accounts utilised emotion strongly within the narrative they had constructed of their experience during their periviable labour; “*You know, it's not a night we'll forget very easily. It was, it felt like we were battling the hospital.”* [Parent of 22 week surviving baby].

Narratives from both professional and parents varied in their presentation of risks over short and long-term timeframes. Some parents focused in on the immediate period and their priority to ensure their child received intensive care; “*….because we need to know that if something happened that night that NICU were there to save our baby*.” [Parent of surviving 22 week baby] and “*….we know if we get to 22 weeks she's got a chance here*” [Parent of 22 week surviving baby]. Other parents reflected that they had wanted more detailed discussion about the potential future complications of periviablity as they tried to picture what the long-term implications could be for them and their baby; “*I think sometimes you want to see different outcomes, you don't just want to see the positive ones, you also want to see the more difficult ones as well just so you can get an understanding of what you are going into*.” [Parent of 23 week surviving baby].

Parent responses also highlighted the rigid use of time by perinatal professionals when determining management, which was perceived by some parents as arbitrary and lacking in empathy for their individual situations; “*….and she [1st Obstetric Consultant] said right…* *23 weeks is the rule of when we can start giving steroids…but she [2nd Obstetric Consultant] knew our history. She said I'm going to give you the steroids, but I'm not going to give them to you until you reach 22 weeks, which was another 24 h or so. So I did kind of go, can you give me them now, please?*” [Parent of 22 week surviving baby], and, “*Some [professionals] were saying they can save babies at 22 weeks…we save babies at 23 weeks and then someone else was telling me 24 weeks.”* [Parent of 23 week surviving baby]. This was echoed in responses from midwifery professionals who confirmed variation in practice based on gestational age (GA) cut-offs, acknowledging this is particularly problematic in the periviable period given the variability in determining the GA with antenatal scans, which can be +/- five days (23); “*I looked after someone and [NICU] said her baby wasn't going to survive at 23 weeks….* *but today we're saying that someone a day later [23* *+* *1 weeks], especially when scans aren't always the most accurate, [NICU] are happy to fully resuscitate this baby* *…* *I just think that's a bit confusing*.”. These quotes exemplify the friction created between professionals' attempts to adhere to quantitative measures to guide decisions made in varied, emotive and highly individual circumstances. Further, the interview data demonstrated that in cases where parents opted for their baby to receive survival-focused care there was potential for disconnect between parent and professional interpretations of the emotive phrase—“do everything”. Whilst this phrase could encompass a range of interventions from simple mask ventilation through to chest compressions and emergency medications being provided at delivery, there was a sense of realism from the parent data which did not place an insistence on specific interventions at birth, but rather, an encouragement of professionals to do what they could to support the baby's survival:

“They [the NICU doctor] did mention something like obviously if she's breathing they will help her and stuff like that and then if she wasn't then that would be it … but I just remember saying just do everything that you can.” [Parent of 23 week surviving baby]

“….it probably was just enough that they [NICU] were going to come and they were going to do something. What that something was going to be, I knew depended on the baby, but we didn't know a lot of what they were going to do.” [Parent of 22 week surviving baby].

Data from the consultant interviews suggests an acceptance of responsibility for the specifics of the extent of stabilisation measures implemented at the birth, whilst placing emphasis on the parental role in making the wider, overarching decision about the overall direction of management for their baby, be that survival-focused or comfort care: “ *…there are those parents who usually want everything doing insofar as—when I say ‘everything’ doing…what I actually mean by ‘everything’ is to offer [breathing] support at delivery and see how they respond.*” [Consultant Neonatologist].

## 5 Discussion

This thematic analysis of parent and professional perspectives within the pre-birth periviable conversation has identified that the roles of time, uncertainty and transparency serve critical functions in determining the alignment of parent and professional within the encounter, their ability to distil a decision regarding the baby's management and the narrative of care going forwards. Malalignment of these three themes can create disconnect and potential for conflict within these vital conversations.

### 5.1 Individualisation of information

The interview responses identified areas of disconnect between parent and some professional definitions of an “individualised” approach to information-sharing. Parents indicated a preference for professionals to have knowledge of their and their baby's unique story and circumstances and apply this information to inform and individualise (where possible) risk stratification of options and potential outcomes. This aligns well with the use of risk stratification profiles from professional frameworks which use holistic assessment of baby's characteristics, such as gestational age, predicted weight, sex, place of birth and if there is time for antenatal steroids, to help inform the level of risk of mortality and guide management at birth for the individual infant (1).

However, within the interview data there were also descriptions of deliberate information selection. These related to professionals determining when to restrict parental access to information, for example, through omission of relevant management options. This occurred in relation to avoidance of discussing the option of survival-focused care for 22 week infants, and, conversely, omitting the option for comfort care for high risk 23 week infants. In these instances, professional frameworks would classify these two groups of infants as “Extremely High” risk and would recommend open discussion of both survival-focused and comfort care with parents. This approach of selective information sharing with parents through omission of relevant management options as determined by the professional, was described by one consultant as individualising information; “It's individualised depending on what you know the parents want to happen”. This is not individualising information and prevents parents from being fully informed and meaningfully engaged in decisions relating to their baby. As one midwife remarked, “You're making assumptions that you're telling them what you think they want to hear and that's not necessarily the case.”. Information selection serves to restrict parents access to information and autonomy in determining the relative importance of that information to them. Maternity legal cases have emphasised the importance of patient's themselves determining which risks and outcomes, relevant to their care, are important to them and their own decision-making process. Perinatal professionals have a duty to inform patients of all material risks, rather than all those risks determined to be relevant by the professional [see Montgomery vs. Lanarkshire Health Board (24)]. Omitting relevant and applicable management options reduces transparency and may create distrust between parents and professionals.

### 5.2 Respecting the role of the parent

The issue of meaningfully individualising care extends to a specific issue of language use within these pre-birth periviable conversations. Professional interview responses included references to parents requesting “everything be done” for their baby. Within the traumatising context of periviable birth and with parents, as described in the interviews, potentially receiving care from perinatal professionals with an overtly pessimistic approach toward periviable birth and outcomes, there may well be a natural desire from parents for their baby to have access to all available treatment and to be given the best chance of survival. The role of the parent to advocate on behalf on their child is a well-established narrative and one that parents need to be empowered within to fulfil their desired role as a “good parent” to their baby (25, 26). The good parent role describes the desires of parents of critically ill and dying children to be able to fulfil several roles within their child's care and aids parental decision-making and coping. These include being informed about their child's care, being able to reduce their child's pain and ensuring that their child feels loved. In the face of often overt pessimism from perinatal professionals, parents in periviable labour may assert their parental role by requesting “everything” be done for their baby. This desire should be respected by perinatal professionals, but this does not mean this assertion does not require further exploration. This assertion, to “do everything” from parents does not equate automatically to insistence that their baby receives chest compressions. Indeed, within this interview cohort parents wanted balance between optimising their baby's chance of survival against avoiding their baby being in pain or suffering. For some parents, their desire for “everything” to be done extended to antenatal optimisation and neonatal attendance at delivery with an acceptance that their baby may not respond to airway support and may die in the delivery room; “[It]…was just enough that they [NICU] were going to come and they were going to do something. What that something was going to be, I knew depended on the baby”. Discussing management options with parents utilising parallel planning principles to outline and describe different potential outcomes for baby is important in empowering parents within their parental role and implementing trauma-informed care principles of choice and control. This approach, of transparent discussion of all potentially relevant management options at delivery through parallel planning structure, can establish a level of trust between parent and professional and may avoid development of combative narratives with parents feeling the need to “fight” for access to information and treatment.

Awareness of the role that narrative plays in information processing and decision-making can help professionals attune to where parents are within their personal life narrative and enable professionals to better support, rather than antagonise, parents. Human beings are innately storytelling creatures whose brains have evolved to use narrative to predict, inform and adapt to our physical and social environments (27). Everyone, parent and professional, has their own narrative arc that they are travelling along in life. Others come into, and out, of the narrative and occupy distinct roles within how we understand and make sense of events in the present and over time (27). For parents, the event of periviable birth presents an unwelcome rupture to their imagined future and is a significant incongruity within their personal narrative. In response to significant interruptions in the narrative, particularly interruptions associated with significant uncertainty, such as facing periviable birth, individuals need information with which to mentally run a series of simulated (or imagined) futures, in order to determine the most appropriate course of action to take for them. Emotions serve as an important tool for prioritising these simulated (or imagined) futures, as seen in the varied parental responses to hope and uncertainty in the interview data.

**Figure 2:**
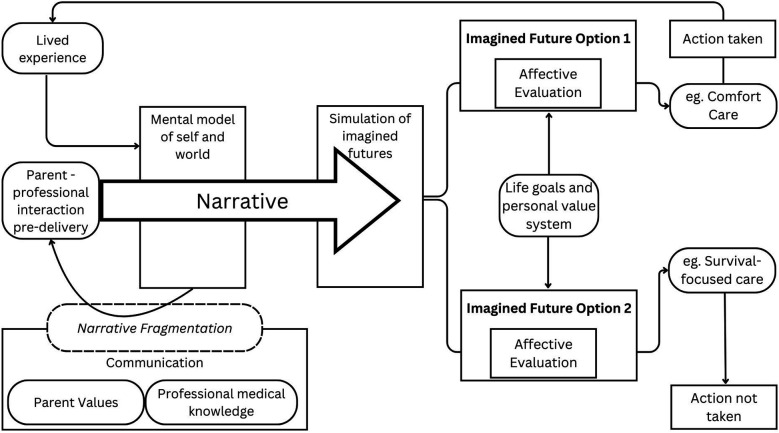
Cognitive narrative theory applied to periviable birth decision-making [adapted from Johnson model (28)].

Conviction Narrative Theory (CNT) outlines this human approach to decision-making (28). CNT is a theory of choice under conditions of radical uncertainty which refers to situations where the probabilities of all potential outcomes cannot be assigned (29). Radical uncertainty applies to periviablity where even with the current risk stratification tools, it is not possible to accurately predict the outcome for an individual infant. Under these intensely uncertain conditions, the brain utilises narratives (structured representations of causal, temporal, analogical, and valence relationships) in place of probabilities, within decision-making processes. CNT describes the process by which the brain will construct narratives based on individual understanding and the surrounding social environment and use these narratives to construct a series of imagined futures. The brain will run through these imagined scenarios, using the triggered emotional reaction that the scenario evokes as a means of evaluating and prioritising each potential scenario (29). In this way the individual adopts the narrative that “feels right” to explain their situation (available data) and select the best imagined future to use to make their choice (affective evaluation) (Figure 2). The cognitive narrative theory model accounts for the different responses to uncertainty and risk that were illustrated within the interview responses; for example, parents reporting the that regret they would experience from not trying survival-focused care would exceed the imagined regret they may experience from their baby going through intensive care.

It is important that within these pre-birth conversations, professionals are cognisant there are varied personal narratives for each person (parent and professional) involved in the discussion. This can mean that each person within the conversation is at a different point in their own narrative around periviablity and within the overall narrative of that individual baby, and this can give rise to disconnect and conflict. Professionals are balancing their own considerations of immediate management priorities and future risks and likelihoods to discuss with parents, their experiences caring for previous periviable patients and navigating their own moral values. Parents may be balancing their desire to advocate for their baby, fear for their baby's future, fear of suffering for their baby, uncertainty over the future for themselves, their other children, future pregnancies, their wider family, career and life. Each of these considerations requires mental prioritisation and alignment with the individual's self-narrative (the narrative the individual has about what kind of person they are). Applying CNT, whilst professionals may prefer to consider themselves rational, logical decision-makers, they are fundamentally human beings and therefore, the inherent uncertainty involved in periviable birth will prompt CNT processes for them too. Professionals will utilise their clinical knowledge and experience, integrating this alongside their emotive experiences of the care they have provided to previous periviable infants, in order to run mental simulations of multiple possible outcomes. These may include performing survival-focused care and the baby surviving with no complications (the healer), survival-focused care being implemented but being ultimately unsuccessful and having performed multiple invasive procedures on a dying baby (the torturer), or the most uncertain of all, advocating for comfort care at birth and not knowing if the baby could have survived had intensive care been implemented. Parents will also be running their own imagined futures, prioritising their decision-making through affective evaluation of the options presented to them: opting for survival-focused care and being a parent of a surviving child; being a parent to a disabled surviving child; being a bereaved parent.

It is essential that parents are permitted to run these imagined scenarios through in their mind and that the role that emotion plays within decision-making in these radically uncertain circumstances is respected and not diminished. Perinatal professionals need to be able to be reflexive within their professional role by being aware of their own biases and emotions related to their previous periviable experiences that they may be bringing into the conversation. Professionals need to be equipped to maintain insight into their own emotion-driven processes within these high-stakes decisions and be able to facilitate parents through theirs. Reflective practice in these circumstances means professionals being able to actively listen to where parents are within their narrative and flexibly provide information matched to the parents' thoughts and questions, allowing them to construct, run and prioritise the necessary imagined future scenarios. Parallel planning discussions can be a useful tool in outlining multiple potential outcome scenarios with parents in a structured progression, enabling transparent information-sharing, acknowledging the parental role and empowering parent choice within an inherently traumatic and uncertain period in their lives.

### 5.3 Clinical implications

**Figure 3:**
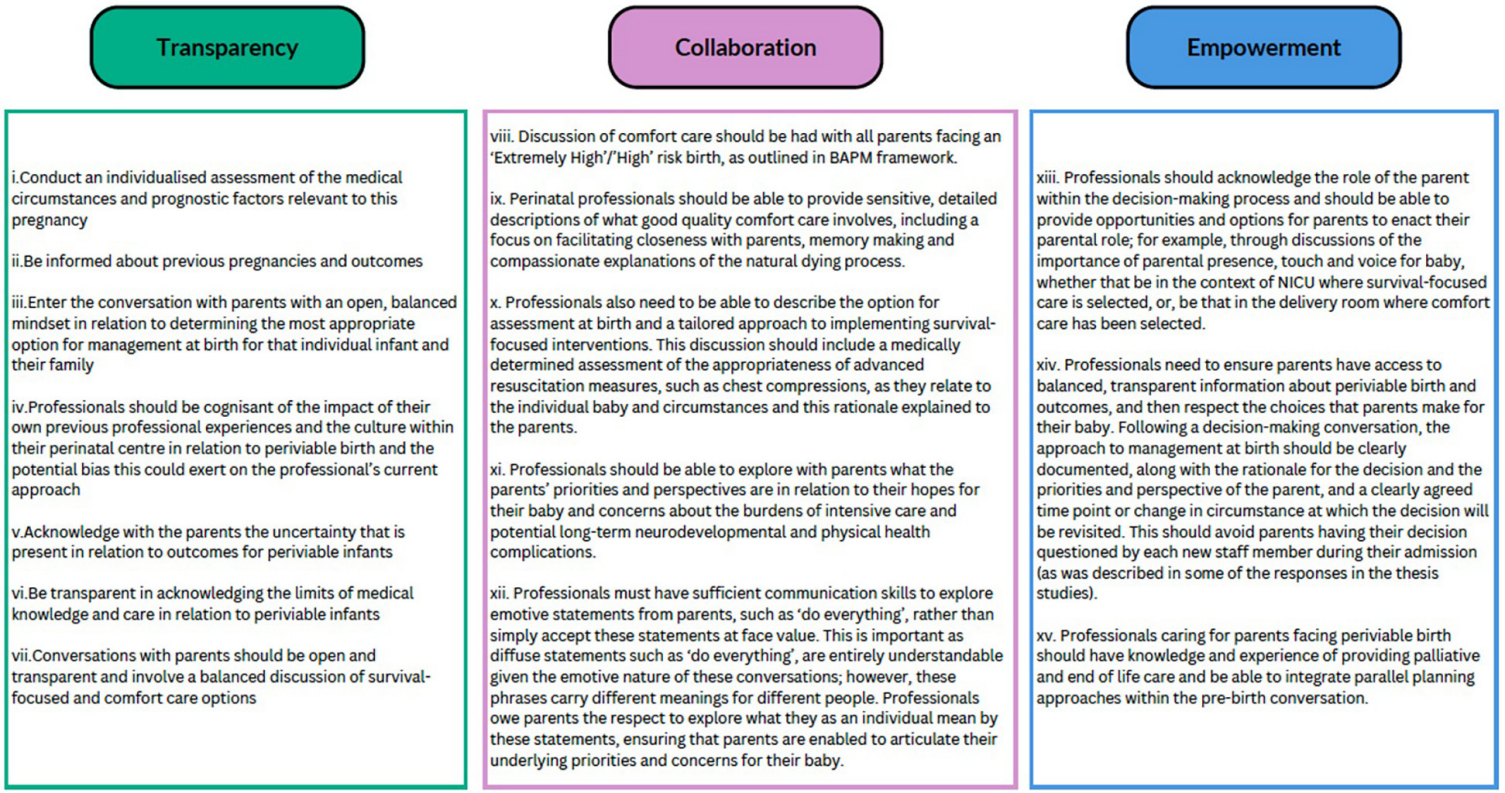
Recommendations for a trauma-informed approach to the pre-birth periviable conversation for perinatal professionals.

Within the periviable pre-birth conversations, the quantitative evidence base that informs professional guidelines will continue to evolve and the underlying statistics for survival and likelihood of complications will continue to be updated as knowledge expands and medical management of the periviable infant is optimised. The overall approach to conveying these potential outcomes and management options to the individual parents facing periviable birth within each pre-birth conversation can be improved and refined with adherence to the findings of this study: the need to understand narrative within decision-making, awareness of reactions to uncertainty, the need for transparency and the need to empower the role of the parent. Communication built from a basis of compassion and narrative competence is required not only within the pre-birth periviable conversation, but across perinatal care in general. The findings from this study can be integrated within a trauma-informed care approach which could be used by professionals to improve care delivered to future families (Figure 3).

### 5.4 Strengths and limitations

This study is the first in-depth exploration of parent and perinatal professionals' perspectives on the pre-birth periviable conversation. The study recruited parents with a range of lived experiences, ethnicities and socioeconomic backgrounds. All perinatal professional groups involved in pre-birth periviable decision-making conversations and providing care to parents facing periviable birth were represented in the interview data. The interviews have gathered rich data and provided detailed considerations for clinical practice and future research.

The study is limited to one geographical area within the North West of England which may impact the generalisability of the results. Additionally, despite the efforts of the research team to include this group within the recruitment protocol, there were no parents who had elected for comfort care at birth. Some of the parents included in this study had experience of birth between 21 + 0–21 + 6 weeks gestation. The research team did discuss inclusion of comfort care within the pre-birth conversation with the recruited parents.

## 6 Conclusion

This study has gathered perspectives on the pre-birth periviable conversation from perinatal professionals and parents with lived experiences of periviable birth. The interview data identified that the three themes of narrative, uncertainty and transparency of information play key roles within these pre-birth conversations and decision-making processes. From these three themes, strategies to improve future pre-birth conversations were established and a set of recommendations for improving future conversations was developed using a trauma-informed framing. The recommended approach highlights the need to empower and respect the role of the parent and the role of narrative decision-making within these pre-birth conversations. Professionals can empower this parental role through transparent information provision, outlining all relevant management options with parents and not selectively omitting applicable options based on professional discretion. Understanding the role of emotion and narrative within decision-making in situations of significant uncertainty, such as periviable birth, allows the professional to appreciate, rather than discount, the relevance of parent emotion and facilitate them to participate in these critical decisions for their baby.

Future research is required to better understand the educational methods best suited to train perinatal professionals to incorporate these, and other trauma-informed care principles, within their communication and interactions with future parents.

## Data Availability

Anonymized data will be available on reasonable request to the corresponding author.
