# Supplementary material for: Parent and perinatal professional priorities and perspectives for the pre-birth periviable conversation: a thematic analysis of semi-structured interviews
Source: Front Pediatr. 2025 Jul 1;13:1552911. doi: 10.3389/fped.2025.1552911 (PMC12266255; doi:10.3389/fped.2025.1552911)
Supplement: Supplementary file 1 [file Supplementaryfile1.pdf]

PeriviAble DeLiveries: ALIgning PArental aNd PhysiCian PrioritiEs (ALLIANCE)

Semi-structured Interview Themes:

The following are examples of questions that would be included in each interview question theme. The interview will be responsive to the answers the parent or clinician give, and to the emotional state of the interviewee.

PDPVC = Pre-Delivery PeriViable Conversation

Before the interview is started:

1. Thank the participant for agreeing to be interviewed. Introduce self and role.
2. Everything you say in this interview will be anonymised and your confidentiality maintained.
3. Ask if they are still OK for me to tape record this conversation.
4. Ask if they have any concerns before I start.
5. Remind them that it is OK to stop at any point or refuse to answer any questions during this interview.
6. Restate that the purpose of this research is to explore parental perspectives on the conversations they had with healthcare professionals before the baby was delivered about the prognosis and management options/strategy.
7. Tell them that there are 4 sections (parents)/ 3 sections (clinicians) to this interview and 4 closing statements.

Parent Interview Themes: I would work through Sections 1 and 2 as able with each parent; if time and emotional state of the parent permits I would then move into questions in Sections 3 and 4.

1. The Delivery and Child

- Tell me about your child/baby/delivery?
- (For survivors) How are they doing now?
- What do they enjoy doing? Anything they need extra help with?
    - Ask about school, nursery, interaction with siblings as appropriate.
- What's it been like parenting an extremely premature child? (Impact on them and family).
- What's it been like experiencing the loss of an extremely preterm baby? (Impact on them and family).

2. The Discussion

- Do you remember meeting with the medical staff before the delivery?
- What do you remember about these conversations?
- Who did you meet with?
- What did they discuss with you about the situation, the delivery, the baby?
- How did you feel about having these pre-delivery conversations?

- How did you feel during these pre-delivery conversations?

- What then happened during your labour/delivery/NICU stay?
- With the experience and knowledge you have now, what do you think the healthcare team should have told you?
- Do you think they should have explained anything more or less?
- What did they not mention that they should have?
- Was there anything that was discussed that should not have been?

- Were the long-term complications of prematurity discussed?
    - any discussion about what happens if baby survives to discharge?
    - any discussion about the impact on yourself, as a couple, as a family?
    - any discussion about your family structure and support options?
- Did the team give indications of the range of outcomes that periviable infants face?
- Have you heard the term comfort care?
- Was comfort care discussed with you as an option?
    - If so, at what stage in the discussion?
    - How was it described?
    - How did you feel about it being discussed?
    - Would you have wanted this to be part of the discussion?
- Was potential pain for the baby from resuscitation/procedures on NICU discussed with you?
    - How was this raised and discussed?
    - What do you think about this being discussed with parents?

3. Reflections on the conversation

- Did the conversations you had pre-birth manage to prepare you for the delivery/for NICU?
- What things do you think should be discussed during the PDPVC?
- Do you think the clinical team were in agreement with the options they presented?
- Do you think it is appropriate for the clinical team to offer their management recommendation based on their clinical judgement to parents?
- Did you feel you had a say in making the decision?
    - Did you find being involved in making the decision helpful?
    - Did you find helping with/making the decision too much?
    - If parent did not feel they made the decision, then, if able, ask about whether they think they would have found this preferable?

4. Improving the process

- What key things would you want parents to know if they went through a similar experience to yourself?

- How do you think medical staff could improve preparing parents for periviable delivery?
- What pregnancy resources did you use in your pregnancy? If any?
- Was preterm delivery discussed with you during your pregnancy before you went into labour?
- Do you think it would be appropriate to share information about periviable and preterm births earlier in the pregnancy? If so, how much?
- Would you have had discussions with your partner about things like extreme preterm birth before it happened it you?
- Would information about preterm birth during your pregnancy be helpful? Or stressful?
- What level/type of information should be available?

Clinician interview Themes: I would work through sections 1 and 2 with each clinician; if time and emotional state of the clinicians permits, I would then move into Section 3.

1. Experience with PDPVC's

- How recently was your last PDPVC?
- How many PDPVC's have you been involved in?
- What's the lowest gestation you have had this type of conversation?

2. The Content

- How do you approach these conversations?
- Do you feel these conversations are similar or different to usual clinical conversations you have with parents?
    - How?
- Do you have certain techniques or structures for these conversations?
    - If so, can you explain these to me?
    - If not, why not?
- Topics covered (short and long term and order these are presented)
- Does how you approach the PDPVC vary according to gestation?
    - If so, how?
- How do you deal with the uncertainty in our knowledge of outcomes at these gestations?
- Do you explain this uncertainty to the parents?
- Do you vary your counselling depending on how imminent labour is?
- How does it vary?
    - imminent delivery (<30 mins to deliver)
    - delivery likely within next few hours
    - presented with history of contractions. SROM, 2-3cm dilated and settling.

- What key messages do you convey to the parents?
- What information do you require from them?
- Do you offer the option of comfort care?
- How do you explain/introduce this?
- Which contexts would you offer this in?
- Do you think intensive care involves pain for these infants?
    - Do you discuss the issue of intensive care related pain during these PDPVC's?
        - If so, how and in what contexts?
        - If not, why not?

3. Improving the Discussion

- What constitutes a 'good' PDPVC for you?
- Why?
- How do you evaluate your own performance in PDPVC's?
- Do you sit in on other clinician's counselling?
- Do you think current practices around PDPVC's are optimal?
- If not, why? How would you improve things?
- How would you know this was an improvement?
- Do you think the concept of preterm birth should be introduced to parents or discussed earlier in the pregnancy?
- What effect (if any) do you think this might have on PDPVC's if they then happen subsequently?

Closing Statements for both groups of Interviewees:

- Is there anything that we haven't covered already that you would like to talk about before we finish?
- Remind the participant:
    - That everything they say in the interview will be anonymised and confidentiality maintained.
    - That they can withdraw if they choose by simply contacting the research team.
    - Ask if they have any questions.
    - Thank them for their participation.
