# Supplementary figures and images for: Parent and perinatal professional priorities and perspectives for the pre-birth periviable conversation: a thematic analysis of semi-structured interviews
Source: Front Pediatr. 2025 Jul 1;13:1552911. doi: 10.3389/fped.2025.1552911 (PMC12266255; doi:10.3389/fped.2025.1552911)

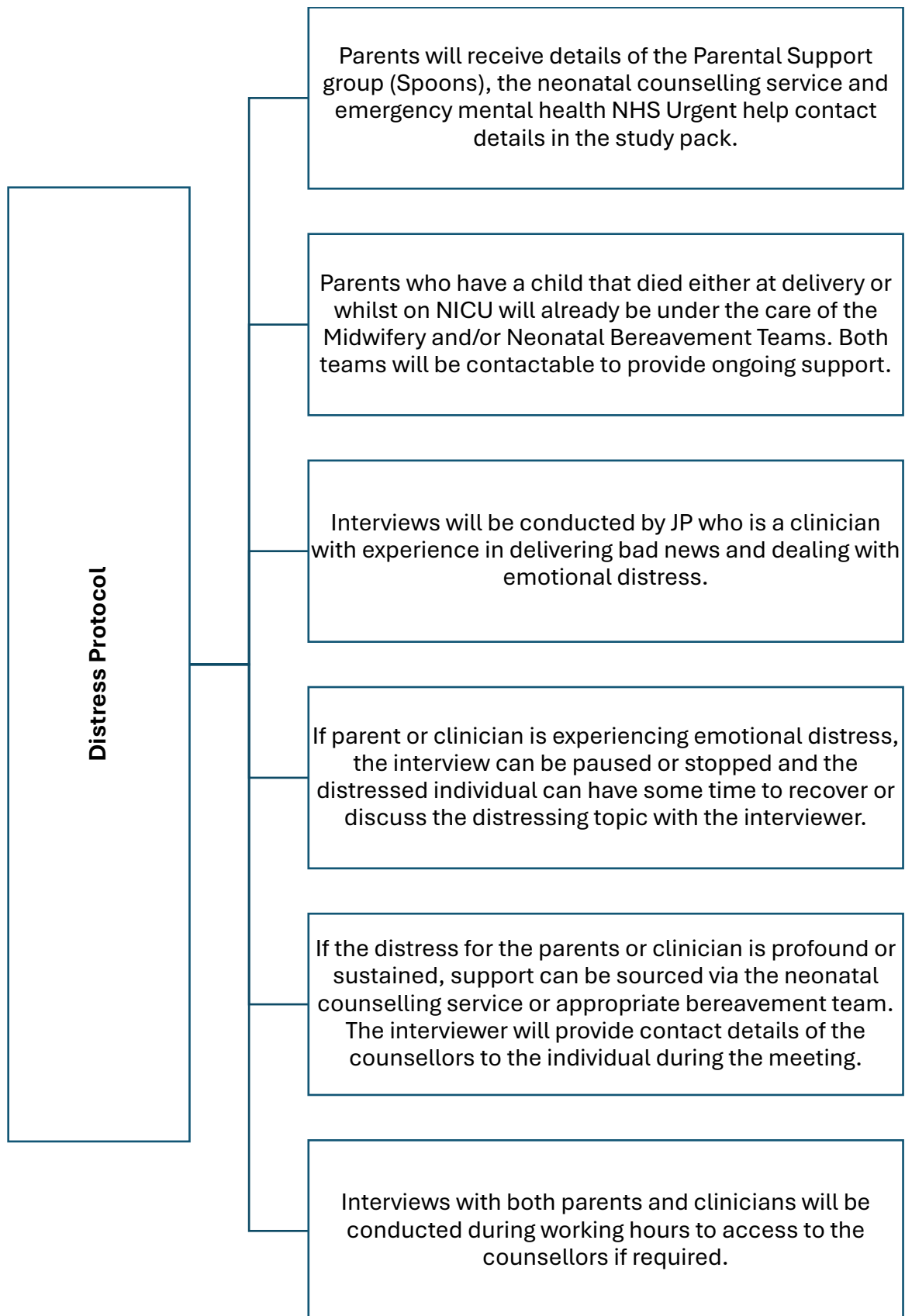

**Supplemental Material: Distress Protocol**

Supplement: Supplementary file 2 [file Supplementaryfile2.pdf]
